# Characterization of Polyphenolic Compounds Extracted from Different Varieties of Almond Hulls (*Prunus dulcis* L.)

**DOI:** 10.3390/antiox8120647

**Published:** 2019-12-16

**Authors:** Maher Kahlaoui, Stefania Borotto Dalla Vecchia, Francesco Giovine, Hayet Ben Haj Kbaier, Nabiha Bouzouita, Letricia Barbosa Pereira, Giuseppe Zeppa

**Affiliations:** 1 Department of Agriculture, Forest and Food Sciences (DISAFA), University of Turin, Largo Paolo Braccini, 2, 10095 Torino, Italy; stefania.borottodallavecchia@unito.it (S.B.D.V.); francescogiovine7@gmail.com (F.G.); letricia.barbosapereira@unito.it (L.B.P.); 2 Higher School of Food Industries of Tunis (ESIAT), University of Carthage, 1003, 58 Alain Savary, Tunisia; h.kbaier@gmail.com (H.B.H.K.); bouzouita.nabiha@gmail.com (N.B.); 3 Department of Analytical Chemistry, Nutrition and Food Science, Faculty of Pharmacy, University of Santiago de Compostela, 15782 Santiago de Compostela, Spain

**Keywords:** almond hull, *Prunus dulcis L*., ultrasound-assisted extraction, response surface methodology, radical scavenging activity, polyphenols

## Abstract

Ultrasound-assisted extraction (UAE) was applied as a pretreatment technique to improve the recovery of polyphenols from the almond hulls of four Tunisian and three Italian almond varieties, followed by the characterization with HPLC-DAD. The operating parameters (solid/liquid ratio, extraction time, and ethanol concentrations) were optimized using a Response Surface Methodology. A polynomial equation was calculated to describe the relationship between the operating parameters and dependent variables as total polyphenolic content (TPC) and antioxidant activity (RSA). A desirability function approach was used to determine the optimum conditions for operating parameters: a solid:solvent ratio of 2 g/100 mL, an extraction time of 13 min, and an ethanol concentration of 51.2%. Among the almond varieties, Pizzuta and Fakhfekh showed the highest polyphenol content and antioxidant activity. HPLC-DAD analysis of almond hull extracts confirmed that chlorogenic acid, catechin, and protocatechuic acid were the most important polyphenols in almond hull. The results highlighted that UAE could be an effective technique for the recovery of phenolic compounds from almond hull, thereby making this byproduct a promising source of compounds with potential applications in food and healthcare sectors.

## 1. Introduction

Almond (*Prunus dulcis* (Mill.), *Prunus amygdalus* (Batch), or *Amygdalus communis* (L.)), is a deciduous tree native to Iran, its surrounding countries, and Central Asia, but is widely cultivated elsewhere under different climates and growing conditions. The almond tree belongs to the *Rosaceae* family, genus *Prunus* with peach, plum, apricot, cherry, and nectarine, a group of fruits collectively known as “stone fruits” i.e., fruits with a stony endocarp. The United States of America, and in particular, California, is the main producer of almonds globally, with a production of about 1.02 million tons in 2017, compared with the worldwide production of about 2.2 million tons in the same year [1]. The edible part of almonds, the kernel, is a seed with two large cotyledons. In the last several years, market demand for almonds as edible nuts and as an ingredient in manufactured food products has significantly increased due to the physico-chemical, nutritional, and sensorial features of this fruit. Indeed, almond kernel is consumed worldwide raw, cooked or dry-roasted, sliced or whole, and blanched (without the skin) or unblanched (with the skin). It is extensively used in bakery and confectionery products and in food preparation in general, but also in pharmaceutical and cosmetic applications. The growing interest in almonds is based on their richness in lipids (about 50% of kernel weight), proteins (16–22% of the kernel weight), dietary fiber (10.8–13.5% of the kernel weight), and minerals [2], as well as their high concentrations of phenolic compounds including benzoic and cinnamic derivates, flavonols, anthocyanidins, and procyanidins [3]. Recent studies have evidenced their beneficial effects due to the capacity of their bioactive nutrients and nonnutrients to lower the plasma level of low-density lipoprotein cholesterol and the incidence and severity of cardiovascular disorder [2].

Unfortunately, almond seed production generates large amounts of byproducts, the heaviest of which is the hull, accounting for 35–62% of the total fresh weight of almond [2].

Generally, almond hulls are considered to be of low economic value, and are used as livestock feed and fuel [2]. However, if fresh, they can also have culinary applications, in addition to many medicinal properties when integrated into a diet, as they are thought to reduce cholesterol levels [4].

Almond hulls are rich source of triterpenoids (betulinic, urosolic, and oleanolic acids) flavonol glycosides, phenolic acids, catechin, protocatechuic acid, vanillic acid, and other polyphenolioc compounds; therefore, this byproduct may be an interesting source of natural antioxidants and other bioactive compounds [4,5,6]. Moreover, the extraction of these products from almond residues could contribute to a reduction in the environmental impact and provide added value to the overall almond production.

There are many techniques for recovering phenolic compounds from plant and vegetable materials, but the classic extraction techniques for bioactive compounds (e.g., maceration or simple agitation) generally require long durations, along with high solvent quantities and high temperatures [7], thus risking the thermal degradation of most bioactive compounds [8,9]. Consequently, to reduce the solvent consumption and extraction duration while increasing the extraction yield and purity of extracts, novel, efficient, and advanced extraction methods have been developed, including super critical fluid extraction (SCFE), ultrasound-assisted extraction (UAE), and microwave-assisted extraction (MAE). In particular ultrasound-assisted extraction relies on acoustic cavitation, which causes disruptions of the cell walls in plant materials, resulting in an extensive release of internal cell compounds and a relatively homogeneous system [10,11]. Since this technique is inexpensive, simple, and effective at the industrial scale [12], several studies have investigated the application of UAE in order to extract bioactive compounds from plants materials also using various novel extractants [13,14,15,16,17].

To the best of our knowledge, no study has reported the application of UAE for the extraction of polyphenols from almond hull. Therefore, the objective of the current study was to characterize, for the first time, the recovery of polyphenolic compounds from the almond hulls of Italian and Tunisian varieties by applying UAE. The effects of different processing parameters of UAE (solid:solvent ratio, extraction time and ethanol concentration) on polyphenol recovery from almond hulls were optimized using a Response Surface Methodology.

## 2. Material and Methods

### 2.1. Chemicals

Folin–Ciocalteu’s phenol reagent, 2,2-diphenyl-1-picrylhydrazyl (95%) (DPPH), 6-hydroxy-2,5,7,8-tetramethylchroman-2-carboxylic acid (97%) (Trolox), sodium carbonate (≥ 99.5%), vanillin (99%), aluminium chloride (99%), sodium nitrite (≥ 99%), (+)-catechin hydrate (>98%), methanol (≥ 99.9%), formic acid (98–100%), and hydrochloric acid (fuming 37%) were obtained from Sigma-Aldrich (Milano, Italy).

All standards for phenolic and organic acids, including chlorogenic, protocatechuic, quercetin-3-glucoside, p-coumaric acids, and caffeic acid were purchased from Sigma-Aldrich (Milano, Italy). Ethanol (≥ 70% and ≥ 99.9%), gallic acid, epicatechin, and sodium hydroxide (1 M) were obtained from Fluka (Milano, Italy). All chemicals were of reagent or HPLC grade, and ultra-pure water was produced using a Milli-Q System (Millipore, Milano, Italy).

### 2.2. Samples

The optimization of UAE was carried out with almond hulls of the Zahaf variety, harvested from the Midwest of Tunisia. The hulls were ground with a ZM200 grinder (Retsch Gmbh, Haan, Germany) to a particle size ranging between 200 and 250 μm. The powder was stored in vacuum-sealed polyethylene bags at 4 °C until analysis.

The optimized UAE process was applied to the hulls of three Italian almond varieties (Fascionello, Pizzuta, and Romana), provided by the “Consorzio della Mandorla d’Avola” (Avola, Italy), and four Tunisian almond varieties (Achaak, Fakhfekh, Laurane, and Zahaf), provided by the Tunisian office of almond and olive oils (Sfax, Tunisia). All samples were ground using a ZM200 grinder (Retsch Gmbh, Haan, Germany) to a particle size between 200 and 250 μm. The powder was stored in vacuum-sealed polyethylene bags at 4 °C until analysis. The dry mater content of the samples was determined at 105 °C using a Gibertini Eurotherm electronic moisture balance (Gibertini Elettronica, Novate Milanese MI, Italy).

### 2.3. Extraction Procedure

#### 2.3.1. Ultrasound-Assisted Extraction (UAE)

The powdered hulls of each almond variety were added to an ethanol/water solution to obtain a 100-mL solution. The solid:solvent ratio and ethanol concentrations were assigned according to the experimental design. Extractions were performed with an ultrasonic bath PEX1-S (REUS, France) at a frequency of 40 kHz and 300 W of power. The extraction duration was also assigned according to the experimental design. The temperature of extraction was 25 ℃, maintained by an external circulation of cold water obtained from a chiller. All the extracts were centrifuged at 5600× *g* for 5 min at 25 °C, and the supernatants were collected and filtered through a 0.45-µm nylon membrane filter. Samples were stored at −18 °C in the dark before analysis. For almond hull characterization, the UAE extractions were done in triplicate.

#### 2.3.2. Conventional Solid–Solvent Extraction (CSE)

Conventional extraction was carried out according to Azadeh [18]. Briefly, 10 g of almond hull powder was mixed with 100 mL of ethanol/water solution (70/30, *v*/*v*), and the extraction was performed at 50 °C for 6 h with a VDRL 711 orbital shaker (Asal S.r.l., Milan, Italy) under constant rotatory agitation at 60 rpm.

All extracts were centrifuged at 5600× *g* for 5 min at 25 °C, and the supernatants were then collected and filtered through a 0.45-µm nylon membrane filter. The samples were stored in amber vials at –18 °C. All extractions were done in triplicate.

### 2.4. Experimental Design

A Central Composite Design (CCD) was followed to allow the fitting of a second-order model, using the Design-Expert software 11.0 (Stat-Ease, Inc., Minneapolis, MN, USA). The CCD consisted of 20 experiments with six replicates at the central point.

All experiments were performed in a random order to minimize the effect of unexplained variability in the observed response due to systematic errors.

The range of each variable was selected according to the results from preliminary experiments, where the effects on the polyphenolic content of hull extracts were assessed for different solid:solvent ratios (2, 2.5, 3.3, 5, and 10 g/100 mL), ethanol concentrations (20%, 40%, 60%, 80%, and 100% *v*/*v*), and extraction times (1, 5, 10, 15, 20, 25, and 30 min), using single-factor experiments. All extractions conditions were done in duplicate. For each extraction, the variables not being studied were kept constant at the central value.

Each independent variable was coded at five levels, i.e., −1.6818, − 1, 0, + 1, and + 1.6818, according to the following equation:

(1) $$X_{i}=\frac{x_{i}-x_{0}}{\Delta x}$$

where *X*_i_ is the coded value of an independent variable, *x*_i_ is the real value of an independent variable, *x*_0_ is the mean of the real value of an independent variable, and Δ*x* is the step change in value.

Response Surface Methodology (RSM) with a Box-Behnken design was employed to determine the optimum levels of solid:solvent ratio (*X*_1_, range: 2 to 5 g/100 mL), extraction duration (*X*_2_, range: 10 to 25 min), and ethanol concentration (*X*_3_, range: 40% to 60%) to maximize the total phenolic content and the antioxidant activity of almond hull extract.

The extraction yield of the total phenolic content (*Y_TPC_*) and the antioxidant activity (*Y_RSA_*) against the three variables (*X*_1_, *X*_2_, and *X*_3_) were evaluated using a polynomial second-order model based on the equation used in response surface analysis to predict the optimum conditions for the extraction process:

(2) $$Y=\beta_{0}+\;\overset{3}{\underset{i=1}{\sum }}\beta_{i}X_{i}+\overset{3}{\underset{i=1}{\sum }}\beta_{ii}X_{i}^{2}+\overset{2}{\underset{i=1}{\sum }}\overset{3}{\underset{j=i+1}{\sum }}\beta_{ij}X_{i}X_{j}+\varepsilon$$

where *Y* represents the predicted response (TPC or RSA values), *X_i_*’s are the level of variables, *β*_0_, *β_i_*, *β_ii_*, and *β_ij_* are the constant, linear, quadratic, and interactive terms, respectively, and *ε* is the error.

#### Verification of the Model and Application

To test the accuracy of the response surface models, the values of total phenolic content and antioxidant activity calculated using the regression model were compared with those obtained from the UAE of hull extracts, carried out under optimal conditions, as determined by RSM.

Finally, the optimal conditions for UAE were applied to almond hull extracts from three Italian and four Tunisian varieties, and the yields were compared against conventional solvent extraction. The extraction procedures were performed in triplicate.

These extracts were also characterized for the levels of flavonoids and condensed tannins, and the most important phenolic compounds were determined by HPLC-DAD analysis.

### 2.5. Analytical Determination

#### 2.5.1. Total Phenolic Content

The total phenolic content (TPC) of the almond hull extracts was determined in triplicate according to the Folin-Ciocalteu colorimetric method, as reported by Singleton et al. [19], with slight modifications. Briefly, 50 µL of extract was mixed with 250 µL of Folin-Ciocalteu reagent and 3 mL of ultrapure water.

The mixture was allowed to equilibrate for 3 min at 20 °C, and then 750 µL of 20% (*w*/*v*) aqueous sodium carbonate solution was added. After incubation at 20 °C for 2 h in the dark, the specific absorbance of the mixture at 765 nm was measured with a UV-Visible spectrophotometer UV-1700 PharmaSpec (Shimadzu, Milano, Italy). A mixture of solvents and reagents was used as a blank. Gallic acid was used as a standard, and the results were expressed as mg of gallic acid equivalents (GAE) per gram of dry sample.

#### 2.5.2. DPPH Radical Scavenging Activity

The free radical scavenging activity (RSA) of the extracts was determined according to the method reported by von Gadow et al. [20] using the stable radical 2,2-diphenyl-1-picrylhydrazyl radical (DPPH), with slight modifications. Briefly, 75 µL of the sample extract were added to 3 mL of 6.1 × 10^−5^ M DPPH/methanol solution and incubated at 20 °C for 1 h in the dark. Afterwards, the decrease in absorbance at 515 nm was recorded using methanol as a control and DPPH/methanol solution as a blank.

The inhibition percentage (*IP*%) of the DPPH by the antioxidant extracts was calculated according to the following formula

*IP*% = [(*AB* − *AS*) / *AB*] × 100 (3)

where AB is the absorbance of the blank and AS is the absorbance of the samples. The results were expressed as µM Trolox equivalents (TE) per gram of dry sample (dw), by means of a dose-response curve for Trolox (0–350 µM).

#### 2.5.3. Condensed Tannin Content

The content of condensed tannins (TCT) in the almond hull extracts was estimated using the method of Lin et al. [21], with slight modifications. An aliquot (50 µL) of each extract was mixed with 3 mL of 4% vanillin in methanol solution, followed by the addition of 1.5 mL of HCl (37% *v*/*v*). The well-mixed solution was incubated at 20 °C for 15 min in the dark. The absorbance of the samples was then recorded against the blank at 500 nm. (+)-Catechin was used to prepare the calibration curve, and the results were expressed as mg Catechin equivalent (CE) per gram of dry sample (dw).

#### 2.5.4. Total Flavonoid Content

The content of total flavonoid (TFC) in the almond hull extracts was determined following the method of Lin et al. [21], with slight modifications. Briefly, an aliquot (300 µL) of each extract was mixed with 1.5 mL of ultra-pure water and 90 µL of 5% NaNO_2_ solution. After 6 min, 180 µL of 10% AlCl_3_ × H_2_O solution was added, and the reaction was allowed to continue for 5 min. After the addition of 1 M NaOH solution (600 µL) and 330 µL of H_2_O, the absorbance against the blank was recorded at 510 nm. (+)-Catechin was used to construct the standard curve, and the results were expressed as mg Catechin equivalent (CE) per gram of sample (dw).

#### 2.5.5. HPLC-DAD Analysis

Chromatographic analysis was performed according to the method described by Barbosa-Pereira et al. [22], with a Thermo-Finnigan Spectra HPLC-PDA system (Thermo-Finnigan, Waltham, USA), equipped with a P2000 binary gradient pump, a SCM 1000 degasser, an AS 3000 automatic injector, and a Finnigan Surveyor PDA Plus detector. The compounds were separated on a Kinetex Phenyl-Hexyl C18 column (150 × 4.6 mm internal diameter and 5-μm particle size) (Phenomenex, Castel Maggiore, Italy) with the thermostat at 35 °C. The two solvents used for the mobile phase were 0.1% (*v*/*v*) formic acid in water (solvent A) and 100% methanol (solvent B). The flow of the mobile phase was set at 1 mL/min and the injection volume was 10 μL. A gradient elution method was applied, starting with a 90% mobile phase composition of (A), maintained isocratically for 2 min. It was reduced to 50% after 30 min, 20% after 40 min, and finally, to 10% after 42 min. PDA spectra were recorded using a full scan modality over a wavelength (λ) range of 200 to 550 nm, and data were quantified using the external standard method with six-point calibration curves. The system and data acquisition were managed by the Chrom Quest software (version 5.0, Thermo-Finnigam, Waltham, USA). For the quantification, a calibration curve was prepared for each identified compound.

#### 2.5.6. Statistical Analysis

The Design-Expert^®^ software version 13 (Stat-Ease, Inc., Minneapolis, MN, USA) was used to perform the Central Composite Design with multiple regression analysis and analysis of variance (ANOVA) of the experimental data.

The adequacy of the fitted model was determined by evaluating the lack of fit and the coefficient of determination (R^2^). The significance of each coefficient was determined using an F-test.

A response surface methodology (RSM) with a Box-Behnken design was used for the optimization of the processing parameters and to study the effect of individual factors and their interactive effects. The polyphenol concentration of hull extracts from Italian and Tunisian varieties was compared by analysis of variance, followed by a Duncan’s multiple range test with Statistica ver. 13 (StatSoft Inc., Tulsa, OK, USA).

## 3. Results and Discussion

### 3.1. Selection range of CCD

The range for each CCD variable (solid:solvent ratio, extraction time and ethanol concentration) was determined based on a preliminary test, where UAE was applied to almond hulls of the Zahaf variety and the TPC yield was evaluated (Table 1).

The TPC of extracts first increased and then reduced with increasing ethanol concentration, with the maximum value (7.69 mg_GAE_ / g_dw_) obtained at 40% ethanol aqueous solution. These results are in agreement with those reported by Pan et al. [23], Spigno et al. [24], and Liu et al. [25], who observed the same effect of ethanol concentration on the TPC of extracts obtained from plant materials. In particular, the TPC values of extracts from black goji berry increased slightly with ethanol concentrations of up to 70%, and then decreased [26]. The maximum value obtained was 17.9 mg_GAE_/g_dw_ at 70% concentration. A similar effect was reported for sunflower seed cake [9], wheat bran [27], black currants [28], and jackfruit peel [29] extracts. This phenomenon could be explained by the fact that the solvent polarity and molecular movement decrease with increasing ethanol concentration, leading to a low dissolution of phenolic compounds via the lowering of the diffusion coefficient and decreased solubility [30,31]. Furthermore, water and low-concentration ethanol can easily pass through the cell membranes, whereas a high ethanol concentration can cause protein denaturation, preventing the dissolution of phenolics [32]. Therefore, ethanol concentrations of up to 60% were suitable for the extraction of phenolics. Moreover, the extraction duration significantly affected the TPC values, and an increase was observed when the extraction duration was increased from 1 to 25 min, followed by a decrease thereafter. These results are in agreement with that of He et al. [26], who reported that the TPC values of extract from black goji berry, obtained by accelerated solvent extractor, increased with increasing the extraction duration until 11 min, and then decreased. As the TPC values increase up to 25 min and a shorter extraction duration also helps reduce energy costs, the 10–25 min range was selected for the RSM trials. Lastly, the TPC values showed no significant difference with changing the solid:solvent ratio, which is in contrast to the findings of Spigno et al. [24]. These authors observed that an increase in the solid:solvent ratio could lead to an increase in the extraction efficiency via increasing the concentration gradient, which causes the mass transfer from the solvent impregnated on the solid particles into the external solvent.

According to the obtained results, the selected range for the solid:solvent ratio was 2 to 5 g/100 mL, for the extraction time was 10 to 25 min, and for ethanol concentration was 40% to 60%. The selected range for each processing parameter and corresponding coded value are reported in Table 2.

### 3.2. Optimization of UAE parameters

Experimental values of TPC and RSA of almond hulls extracts obtained using UAE are shown in Table 3.

The mean TPC values ranged from 6.72 to 8.09 mg_GAE_/g_dw_, while the mean of RSA values ranged from 37.57 to 55.30 µM_TE_/g_dw_.

The use of UAE improved the extraction yield by up to 225% for TPC and 150% for the RSA compared with control tests performed with the conventional solid–solvent extraction. The use of UAE allowed us to significantly reduce the time of extraction, i.e., from 6h to less than 30 min, avoiding the use of temperature that may decrease the extraction yield of polyphenols which are sensitive to degradation at high temperatures, and reducing the costs of the procedure in a potential application at the industrial level.

The data obtained from the central composite design were fitted to second-order polynomial equations, and the significance of the model coefficients was determined by the ANOVA test. The coefficients and corresponding *p* values for each variable are shown in Table 4.

High *F* values (24.55 for TPC and 32.81 for RSA) with very low p values suggest that the fitted model was statistically significant, demonstrating that the TPC and RSA values could be well predicted with the variable ranges used in the model.

The lack of fit was nonsignificant (*p* > 0.05) for both the parameters, confirming that the model adequately reflected the experimental results.

For TPC, the model obtained by RSM had R^2^ = 0.8155, R^2^_Adj_ = 0.7823, and CV% = 2.84. For the RSA, the model had R^2^ = 0.8552, R^2^_Adj_ = 0.8291, and CV% = 4.50. Therefore, a good degree of correlation between the observed and the predicted values was observed for both parameters. Moreover, the low value of coefficient of variation demonstrated a high degree of precision and a good level of reliability of the results.

The quadratic polynomial equations for TPC and RSA (expressed with real values) are presented in Equations (4) and (5), respectively:

TPC = –15.39 + 2.30 *X*_1_ + 0.34 *X*_2_ + 0.64 *X*_3_ + 0.0164 *X*_1_*X*_2_ − 0.0149 *X*_1_*X*_3_ + 0.00125 *X*_2_*X*_3_ − 0.23 *X*_1_^2^ − 0.12 *X*_2_^2^ − 0.00639 *X*_3_^2^ (4)

RSA = −99.86 − 2.12 *X*_1_ + 1.397 *X*_2_ − 6.058 *X*_3_ + 0.346 *X*_1_*X*_2_ − 0.169 *X*_1_*X*_3_ − 0.00789 *X*_2_*X*_3_ − 0.113 *X*_1_^2^ − 0.061 *X*_2_^2^ − 0.0547 *X*_3_^2^ (5)

**Figure 1:**
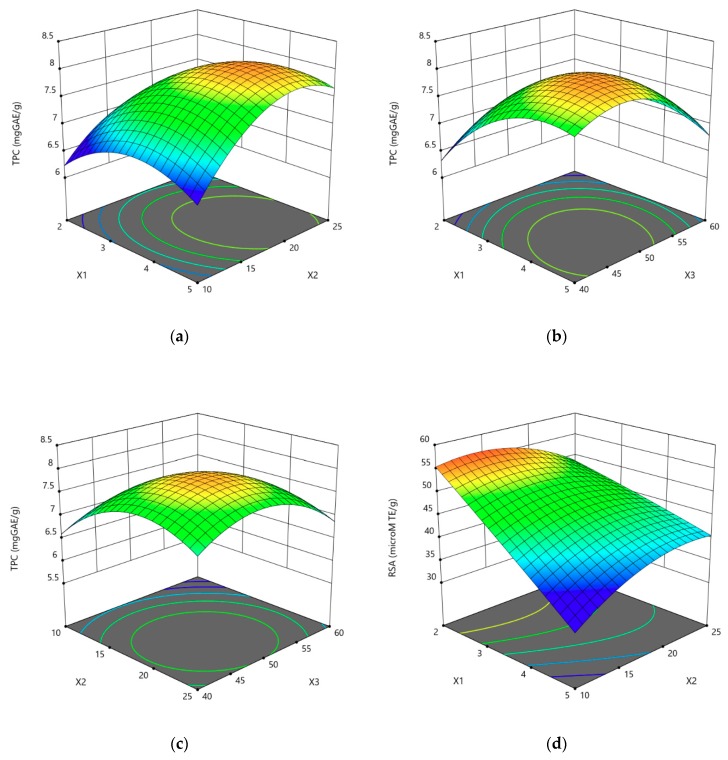

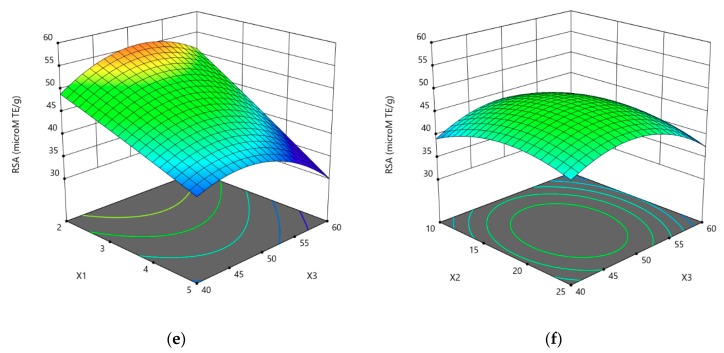
Response surface plots showing the effect of interaction of solid:solvent ratio (*X*_1_; g/100 mL), extraction duration (*X*_2_; min), and ethanol concentration (*X*_3_; % *v*/*v*) on TPC (**a**–**c**) and RSA (**d**–**f**) values of almond hull extracts obtained using UAE. For each graph, the third variable was fixed at the central value.

The solid:solvent ratio, extraction duration, and ethanol concentration had significant linear and quadratic effects on TCP values, while only the solid:solvent ratio and ethanol concentration had significant linear effects on RSA values. The quadratic effects of extraction duration and ethanol concentration are also significant for RSA values. Generally, the interactions between two variables were not significant, except for RSA (*X*_1_*X*_2_ and *X*_2_*X*_3_). The relationships between independent variables and TPC or RSA values were illustrated by a three-dimensional representation of the response surface (Figure 1a–f).

The graphs showed that high values of TPC (Figure 1a–c) and RSA (Figure 1d–f) for almond hull extracts with UAE could be obtained by using low-medium values of solid:solvent ratio and a medium extraction duration using an ethanol concentration of 50%, or by using a low ethanol concentration with long extraction durations. In depth, higher TPC yields can be achieved with moderate–high solid:solvent ratios, using medium–high values of extraction times and intermediate values of ethanol concentration.

These results are in accordance with those reported by Carini et al. [33], Juntachote et al. [34], and Yang et al. [30], who observed that high extraction durations significantly increased the extraction yield of polyphenols, likely due to an increase in the mass transfer of phenolic compounds and a reduction in solvent viscosity and surface tension. Instead, for RSA, higher values were observed using lower solid:solvent ratios and lower extraction times with respect to the TPC. The results highlighted that the main phenolic compounds responsible for the radical scavenging activity of the extracts can be yield under the RSA optimal conditions using lower solid–solvent ratios and lower extraction times. Therefore, under these conditions, more active extracts from almond hulls can be achieved in a sustainable process at room temperature, making it economically feasible for implementation on an industrial scale.

### 3.3. Determination of the Optimal Conditions and Validation of the Model

A desirability function approach of Design Expert ver. 11.0 was used to determine the optimum conditions of solid:solvent ratio, extraction time, and ethanol concentration in order to obtain the highest values for TPC and RSA of almond hull extracts obtained with UAE. The values observed were as follows: solid:solvent ratio of 2 g/100 mL, extraction duration of 13.86 min, and an ethanol concentration of 51.2% *v*/*v*. Under these conditions, the predicted values were 6.81 mg_GAE_/g_dw_ and 56.4 μM_TE_/g_dw_ for TPC and RSA, respectively. A new set of extractions of Zahaf hulls was then performed using UAE, and the obtained experimental values for were 8.30 ± 0.69 mg_GAE_/g_dw_ for TPC and 60.53 ± 2.89 μM_TE_/g_dw_ for RSA, which confirmed that the model was satisfactory and accurate.

### 3.4. Polyphenolic Content and Antioxidant Capacity of Almond Hulls Extracts

The optimized extraction procedure was applied to the almond hulls of three Italian and four Tunisian varieties, and the obtained results were compared with those of traditional solvent extraction.

All almond hull extracts were analyzed for total phenolic compounds (TPC), flavonoids, and tannins, as well as the radical scavenging activity; the results are reported in Table 5.

The use of UAE significantly improved the extraction of phenolics compared to conventional extraction in all almond hull samples studied. The increases in the TPC, TCT, and TFC values observed in the extracts of the various tested samples were up to 2.7–8.2, 5.2–19.0, and 3.5–10.8 times higher, respectively, compared with extracts yielded using the conventional extraction. The increase effect was more evident for samples such as Fascionello, Fakhfekh, and Laurane. The RSA results followed the same tendency as the different groups of polyphenols, and the values observed were up to 2.8–12.4 times higher than the extracts yielded using the conventional extraction. In this case, the Pizutta, Fascionello, and Laurane samples showed a high increase in antioxidant capacity using UAE.

Previous studies have reported that the use of UAE for almond hull extracts improves the extraction yield of polyphenols and the antioxidant activity by up to approximately three times compared with conventional solvent extraction. Examples of these studies include those by Zardo et al. [9], who use the UAE for polyphenol extraction from sunflower seed cake, Hifza et al. [35], who applied UAE for oil extraction, reporting an increment of about 2% compared with conventional Soxhlet extraction, and Ryu and Koh [36], who observed that the highest value was obtained at 18 min for UAE, compared with the conventional solvent extraction duration of 30 min. In the present study, the extraction yield of the different compounds was significantly improved compared to previous studies.

Hull extracts from Pizzuta and Fakhfekh varieties displayed the highest content of polyphenolic compounds (210.49 mg_GAE_/g_dw_ and 207.77 mg_GAE_/g_dw_, respectively), whereas Zahaf showed the lowest (8.37 mg_GAE_/g_dw_) (*p* < 0.05). Pinelo et al. [37] reported that ethanol was the optimal solvent for polyphenol extraction in almond hull; they determined the TPC of 12 almond hull samples using this solvent. The values reported by these authors ranged from 23 to 61 mg_GAE_/g_dw_, which were lower than those obtained in the current study. Subhashinee et al. [38] also found a lower polyphenol content (71.1 mg_GAE_/g_dw_) in almond green cover shell extract using a traditional solvent extraction with 80% (*v*/*v*) ethanol.

The highest flavonoid content was obtained from hull extracts of Fakhfekh (120.04 mg_CE_/g_dw_) and Pizzuta varieties (117.77 mg_CE_/g_dw_), whereas the lowest values were observed for Laurane (77.3 mg_CE_/g_dw_) and Zahaf (3.08 mg_CE_/g_dw_) hull extracts.

A similar trend was observed for condensed tannins, where the highest content was observed for Pizzuta (123.54 mg_CE_/g_dw_) and Fakhfekh (123.49 mg_CE_/g_dw_) extracts, whereas the lowest content was obtained for Zahaf hull extracts (1.78 mg_CE_/g_dw_).

These results highlighted that factors as genotype, cultivation techniques, and climatic conditions may also affect the polyphenolic content in almond hulls, as except for Fakhfekh, the Italian almond varieties showed higher polyphenolic contents than the Tunisian varieties.

The hull samples from Fakhfekh and Pizzuta varieties exhibited the highest RSA values (1990.78 µM_TE_/g_dw_ and 1938.07 µM_TE_/g_dw,_ respectively), whereas Zahaf showed the lowest (64.83 µM_TE_/g_dw_). The hull samples from Fakhfekh and Pizzuta varieties exhibited the highest RSA values (1990.78 µM_TE_/g_dw_ and 1938.07 µM_TE_/g_dw,_ respectively), whereas Zahaf showed the lowest (64.83 µM_TE_/g_dw_).

### 3.5. Identification and Quantification of Polyphenolic Compounds

The HPLC analysis of the extracts highlighted the presence of seven compounds identified by comparison with analytical standards (Table 6). The main phenolic compounds identified and quantified in the several almond hull samples are shown in Table 7. Chlorogenic acid, catechin, and protocatechuic acid were found in all varieties, as were the major phenolic compounds identified in almond hull extracts. Other polyphenols, including quercetin-3-glucoside, p-coumaric acid, and epicatechin were also identified, but only in extracts obtained using UAE, and generally, from Italian almond varieties.

The total amount of polyphenols quantified by HPLC increased from 0.27 to 10.61 mg/g_dw_ as following: Zahaf < Romana < Laurane < Fascionello < Achaak < Pizzuta < Fakhfekh, which confirmed that the differences observed in TPC could be related to the origin of almond. The highest concentrations of phenolic compounds in almond hull extracts were found in Fakhfekh (11. 61 mg/g_dw_) and Pizzuta (7.54 mg/g_dw_) varieties with UAE. In particular, the Pizzuta variety was that with the highest levels of the identified polyphenols, except for catechin and caffeic acid, that were found in higher amounts in the Fakhfekh variety. On the other hand, the Zahaf variety yielded a low polyphenol content, characterized by the presence of only chlorogenic acid, catechin, and protocatechuic acid. The total amounts of the identified polyphenols for each variety were correlated with the TPC, TCT, and TFC values, with correlation values of *r* = 0.7406, *r* = 0.8210 and *r* = 0.7974, respectively, and therefore, with the radical scavenging activity (*r* = 0.6424).

Generally, chlorogenic acid was the most abundant phenolic acid in the almond hull extracts. its content can be correlated (*r* = 0.688) with the antioxidant capacity displayed by the different varieties. Higher correlations were found for TPC, TCT, and TFC, with values of *r* = 0.787, *r* = 0.854, and *r* = 0.826, respectively. Similar results were reported by Takeoka and Dao [39] for almond hull extracts obtained with methanol, wherein the authors identified three hydroxycinnamic acids with reversed phase HPLC-DAD: chlorogenic, cryptochlorogenic, and neochlorogenic acids. Wijeratne et al. [40] also identified flavonols and flavonol glycosides by HPLC as the major flavonoids in the almond green cover shell extracts.

In addition, Sang et al. [41] isolated protocatechuic acid, catechin, urosolic acid, and prenylated benzoic acid derivatives from almond hull extracts. Protocatechuic acid has been described in the literature as one of the major benzoic acid derivatives, with a high antioxidant capacity, and catechin is the most widely-distributed flavonoid in edible plants, with a high antioxidant activity and an inhibitory effect on numerous enzymes [2]. In this study, catechin was the second main compound and the most abundant for the Fakhfekh variety; therefore, it might be also considered as the main compound that contributes to the high antioxidant capacity observed in all of the studied extracts (*r* = 0.5157).

Rubilar et al. [42] reported the occurrence of benzoic and cinnamic acid derivatives, with a small presence of flavan-3-ols, epicatechin, and glycosilated flavonols by HPLC in almond hull extracts.

Wijeratne et al. [40] identified eight phenolic compounds, i.e., protocatechuic acid, quercetin 3-O-rhamnoside, kaempferol-3-O-glucoside, morin, kaempferol 3-O-rutinoside, isorhamnetin 3-O-glucoside, quercetin, and isorhamnetin, from the extracts of whole almond seed, the brown skin, and the hulls.

## 4. Conclusions

To our knowledge, this is the first study describing the polyphenols of almond hulls recovered from different varieties by applying UAE. Using the CCD and the RSM approach, the optimal UAE conditions were determined to enable the extraction of a high quantity of polyphenols from almond hulls, and to obtain the maximum antioxidant activity. We found that a solid:solvent ratio of 2 g/100 mL, an extraction time of 13 min, and an ethanol concentration of 51.2% comprised the optimal UAE conditions. The results highlighted the potential of UAE technology to improve (up to 10 times) the recovery of bioactive compounds from almond hulls as a green extraction alternative to conventional extraction methods with feasible application on an industrial scale. Under these operating conditions, extracts from the hulls of the Fakhfekh and Pizzuta varieties showed the highest contents of polyphenolic compounds, condensed tannins, and flavonoids, and consequently, were also those with highest antioxidant capacity. Chlorogenic acid and catechin were identified and determined in higher amounts for Pizzuta and Fakhfekh hulls, respectively. Furthermore, these compounds were highly correlated with the antioxidant capacity displayed by the extracts yielded from these varieties. Our study highlighted the fact that the content of bioactive components in almond hulls is highly correlated with the variety, as reported in the literature for other vegetable products and byproducts. Finally, although the polyphenolic content in this almond byproduct was lower than in some other byproducts, our results confirmed that the almond hull can serve as a source of multiple bioactive compounds, which could be used not only for innovative and functional foods, but also as ingredients for pharmaceutical compounds.

## Figures and Tables

**Table 1:** Results of single-factor experiments for ultrasound-assisted extraction from almond hull of Zahaf variety and results of ANOVA with Duncan’s test. Values are reported as means ± standard deviation of two extractions.

Solid:Solvent Ratio (g/100mL)	TPC (mg_GAE_/g_dw_)	Extraction Time (min)	TPC (mg_GAE_/g_dw_)	Ethanol Concentration (% *v*/*v*)	TPC (mg_GAE_/g_dw_)
2	6.44 ± 0.25	1	5.05 ± 0.11 ^a^	20	4.47 ± 0.09 ^b^
2.5	6.37 ± 0.44	5	5.81 ± 0.24 ^b^	40	7.69 ± 0.13 ^d^
3.3	6.52 ± 0.46	10	6.22 ± 0.06 ^b^	60	6.52 ± 0.47 ^c^
5	6.28 ± 0.14	15	6.52 ± 0.47 ^c^	80	3.77 ± 0.14 ^b^
10	6.02 ± 0.27	20	6.64 ± 0.13 ^c^	100	0.49 ± 0.01 ^a^
		25	6.87 ± 0.13 ^c^		
		30	6.75 ± 0.29 ^c^		
Significance	ns		**		***

ns, not significant; *** *p* < 0.001; ** *p* < 0.01; mean values in a column with different letters are statistically different for *p* < 0.05.

**Table 2:** Values and coded levels of the independent variables for central composite design used for almond hull extraction.

Independent Variables	Code	Coded Variable Levels				
		−1.6818	−1	0	1	1.6818
Solid:solvent ratio (g/100 mL)	*X*_1_	2	2.6	3.5	4.4	5
Extraction Time (min)	*X*_2_	10	13	18	22	25
Ethanol concentration (%, *v*/*v*)	*X*_3_	40	44	50	56	60

**Table 3:** Composite design and values of total phenolic content (TPC) and radical scavenging activity (RSA) for extracts obtained from almond hull with UAE. Data are reported as means ± standard deviation.

Runs	Uncoded Variables			Responses	
	*X*_1_ (g/100 mL)	*X*_2_ (min)	*X*_3_ (%, *v*/*v*)	TPC (mg_GAE_/g_dw_)	RSA (µM_TE_/g_dw_)
1	2.0	18	50	7.25 ± 0.08	55.30 ± 2.74
2	2.6	13	44	6.90 ± 0.11	50.6 ± 2.09
3	2.6	13	56	6.72 ± 0.11	49.82 ± 2.56
4	2.6	22	44	7.22 ± 0.07	47.54 ± 1.77
5	2.6	22	56	7.28 ± 0.16	49.88 ± 0.31
6	3.5	10	50	7.07 ± 0.05	44.29 ± 0,56
7	3.5	18	40	7.71 ± 0.10	44.56 ± 1.13
8	3.5	18	60	7.02 ± 0.40	39.87 ± 1.35
9	3.5	25	50	7.61 ± 0.15	44.23 ± 2.29
10	4.4	13	44	6.95 ± 0.26	38.0 ± 1.63
11	4.4	13	56	6.95 ± 0.26	37.57 ± 1.63
12	4.4	22	44	8.03 ± 0.06	44.41 ± 1.60
13	4.4	22	56	7.66 ± 0.30	39.19 ± 1.61
14	5.0	18	50	7.71 ± 0.29	39.57 ± 0.76
15	3.5	18	50	7.94 ± 0.21	47.8 ± 2.31
16	3.5	18	50	8.09 ± 0.02	48.00 ± 2.96
17	3.5	18	50	8.01 ± 0.22	47.82 ± 1.62
18	3.5	18	50	8.02 ± 0.21	50.32 ± 1.57
19	3.5	18	50	7.88 ± 0.06	46.09 ± 1.53
20	3.5	18	50	8.06 ± 0.25	46.49 ± 1.89

*X*_1_ solid:solvent ratio (g/100mL); *X*_2_ extraction time (min); *X*_3_ ethanol concentration (%, *v*/*v*).

**Table 4:** Analysis of variance of second-order polynomial models for the total phenolic content (TPC) and the radical scavenging activity (RSA) determined on almond hull extracts obtained using UAE.

	TPC (mg_GAE_/g_dw_)					RSA (µM_TE_/g_dw_)				
	SS	df	MS	F value	P value	SS	df	MS	F value	*P* value
Model	10.04	9	1.12	24.55	<0.0001	1243.40	9	138.16	32.81	< 0.0001
*X*_1_	1.54	1	1.54	33.95	<0.0001	931.50	1	931.50	221.20	< 0.0001
*X*_2_	2.23	1	2.23	49.09	<0.0001	5.33	1	5.33	1.26	0.2661
*X*_3_	0.9285	1	0.9285	20.43	<0.0001	31.52	1	31.52	7.48	0.0086
*X*_1_ *X*_2_	0.1019	1	0.1019	2.24	0.1405	45.50	1	45.50	10.81	0.0019
*X*_1_ *X*_3_	0.1498	1	0.1498	3.30	0.0754	19.45	1	19.45	4.62	0.0365
*X*_2_ *X*_3_	0.0264	1	0.0264	0.5809	0.4495	1.05	1	1.05	0.25	0.6192
*X*_1_^2^	1.48	1	1.48	32.64	<0.0001	0.35	1	0.35	0.083	0.7743
*X*_2_^2^	2.36	1	2.36	52.01	<0.0001	63.55	1	63.55	15.09	0.0003
*X*_3_^2^	2.21	1	2.21	48.58	<0.0001	161.94	1	161.94	38.45	<0.0001
Residual	2.27	50	0.0454			210.26	50	4.21		
Lack of fit	0.4513	5	0.0903	2.23	0.0675	35.76	5	7.15	1.84	0.1240
Pure error	1.82	45	0.0405			174.80	45	3.88		
Cor Total	12.31	59				1453.96	59			

*SS*, Sum of Squares; *df*, degrees of freedom; *MS*, Mean Square; *X*_1_, solid:solvent ratio (g/100 mL); *X*_2_, extraction duration (min); *X*_3_, ethanol concentration (%, *v*/*v*).

**Table 5:** Mean values (*n* = 3) and standard deviation of radical scavenging activity (RSA) and total phenolic content (TPC), total flavonoid content (TFC), and total condensed tannins (TCT) in extracts obtained using ultrasound-assisted extraction (UAE) and conventional solvent extraction (CSE) of almond hull samples. For each analytical parameter, an analysis of variance with Duncan’s test was performed to compare the varieties and the two extraction methods.

	**TPC (mg_GAE_/g_dw_)**			**TCT (mg_CE_/g_dw_)**		
	**UAE**	**CSE**	**Significance**	**UAE**	**CSE**	**Significance**
Pizzuta	210.49 ± 1.79 ^d^	31.98 ± 12.05 ^c^	***	123.54 ± 2.8 ^d^	23.54 ± 11.59 ^c^	***
Fascionello	146.47 ± 5.83 ^bc^	19.78 ± 1.69 ^b^	***	80.21 ± 0.62 ^c^	9.62 ± 0.53 ^b^	***
Romana	160.03 ± 25.44 ^c^	25.66 ± 5.55 ^b^	***	74.36 ± 5.78 ^b^	13.11 ± 3.39 ^b^	***
Achaak	138.70 ± 4.52 ^bc^	24.23 ± 4.57 ^b^	***	81.34 ± 1.01 ^c^	11.30 ± 2.98 ^b^	***
Fakhfekh	207.77 ± 4.23 ^d^	25.79 ± 4.04 ^b^	***	123.49 ± 4.40 ^d^	15.20 ± 2.71 ^bc^	***
Laurane	133.69 ± 5.89 ^b^	16.32 ± 2.21 ^b^	***	70.76 ± 1.33 ^b^	8.73 ± 1.36 ^b^	***
Zahaf	8.37 ± 0.83 ^a^	3.08 ± 0.09 ^a^	**	1.78 ± 0.22 ^a^	0.09 ± 0.02 ^a^	**
Significance	***	*		***	*	
	TFC (mgCE/gdw)			RSA (µM_TE_/g_dw_)		
	UAE	CSE	Significance	UAE	CSE	Significance
Pizzuta	117.77 ± 4.84 ^d^	16.83 ± 7.99 ^c^	***	1938.07 ± 18.69 ^d^	183.62 ± 25.22 ^cd^	***
Fascionello	80.29 ± 4.17 ^bc^	9.07 ± 0.74 ^b^	***	1606.83 ± 62.92 ^c^	142.97 ± 10.44 ^bc^	***
Romana	73.37 ± 2.77 ^b^	12.36 ± 3.56 ^bc^	***	1565.18 ± 42.14 ^c^	168.96 ± 22.90 ^c^	***
Achaak	81.29 ± 4.48 ^c^	11.17 ± 3.14 ^bc^	***	1450.47 ± 131.03 ^b^	167.74 ± 26.50 ^c^	***
Fakhfekh	120.04 ± 4.96 ^d^	12.65 ± 2.52 ^bc^	***	1990.78 ± 39.58 ^d^	203.43 ± 8.72 ^d^	***
Laurane	77.30 ± 3.04 ^bc^	7.19 ± 1.45 ^ab^	***	1607.18 ± 36.74 ^c^	129.49 ± 12.32 ^b^	***
Zahaf	3.08 ± 0.28 ^a^	0.87 ± 0.04 ^a^	**	64.83 ± 3.04 ^a^	23.43 ± 7.36 ^a^	***
Significance	***	*		***	***	

Mean values followed by different letters within the same column are statistically different at *p* < 0.05 (Duncan’s test). *** *p* < 0.001; ** *p* < 0.01; * *p* < 0.05.

**Table 6:** Retention time (Rt), detection wavelength (λmax), linear equation, investigated linear range, determination coefficient (*r*^2^), linear range, LOD, and LOQ of the phenolic compound standards.

Compound	Rt (min)	λ_max_ (nm)	Linear Range (mg/L)	Linear Equation	*r* ^2^	LOD	LOQ
						(mg/L)	(mg/L)
Protocatechuic acid	4.36	293	0.2-202	*y* = 78697*x* + 19672	0.9997	0.1	0.2
Catechin	8.7	279	0.5-194	*y* = 32326*x* − 13902	0.9999	0.2	0.5
Caffeic acid	10.87	325	0.2-23.6	*y* = 234675*x* − 53489	0.9997	0.1	0.2
Chlorogenic acid	11.54	325	0.5-119	*y* = 116431*x* + 47158	0.9996	0.2	0.5
Epicatechin	13.48	279	0.5-198	*y* = 31724*x* + 35810	0.9998	0.2	0.5
p-Coumaric acid	16.34	325	0.2-113	*y* = 178167*x* + 12076	0.9999	0.1	0.2
Quercetin-3-glucoside	24.36	355	0.5-110.0	*y* = 84895*x* + 14596	0.9996	0.1	0.5

**Table 7:** Mean value (*n* = 3) and standard deviation of polyphenolic compounds (mg/g_dw_) identified and quantified by HPLC-DAD in almond hull extracts obtained using UAE and CSE, and the results of the analysis of variance with a Duncan’s test, between the varieties and the two extraction methods.

	**Quercetin-3-glucoside**			**p-coumaric acid**		
	**UAE**	**CSE**	**Significance**	**UAE**	**CSE**	**Significance**
Pizzuta	0.02 ± 0.00 ^b^	nd	***	0.03 ± 0.01 ^b^	nd	***
Fascionello	0.003 ± 0.01 ^ab^	nd	***	0.006 ± 0.01 ^a^	nd	***
Romana	0.006 ± 0.01 ^a^	nd	**	nd	nd	
Achaak	nd	nd		nd	nd	
Fakhfekh	nd	nd		nd	nd	
Laurane	nd	nd		nd	nd	
Zahaf	nd	nd		nd	nd	
Significance	**			**		
	Chlorogenic acid			Epicatechin		
	UAE	CSE	Significance	UAE	CSE	Significance
Pizzuta	4.76 ± 0.25 ^e^	0.12 ± 0.09 ^b^	***	0.03 ± 0.00 ^b^	nd	***
Fascionello	1.60 ± 0.12 ^d^	0.003 ± 0.001 ^a^	***	0.006 ± 0.01 ^a^	nd	***
Romana	0.67 ± 0.24 ^b^	nd	***	nd	nd	
Achaak	1.25 ± 0.08 ^c^	nd	***	nd	nd	
Fakhfekh	3.35 ± 0.07 ^e^	0.006 ± 0.01 ^a^	***	nd	nd	
Laurane	0.92 ± 0.28 ^b^	0.006 ± 0.01 ^a^	***	nd	nd	
Zahaf	0.006 ± 0.01 ^a^	nd	***	nd	nd	
Significance	***	*				
	Protocatechuic acid			Catechin		
	UAE	CSE	Significance	UAE	CSE	Significance
Pizzuta	0.22 ± 0.01 ^b^	nd	***	2.40 ± 0.08 ^d^	0.06 ± 0.04 ^ab^	***
Fascionello	0.07 ± 0.000 ^a^	nd	***	0.51 ± 0.05 ^b^	0.003 ± 0.001 ^a^	***
Romana	0.06 ± 0.03 ^a^	nd	***	0.47 ± 0.09 ^b^	0.01 ± 0.0001 ^b^	***
Achaak	0.04 ± 0.01 ^a^	nd	***	1.51 ± 0.03 ^c^	0.01 ± 0.0001 ^b^	***
Fakhfekh	0.18 ± 0.01 ^b^	nd	***	6.91 ± 0.16 ^e^	0.02 ± 0.0001 ^b^	***
Laurane	0.19 ± 0.01 ^b^	nd	***	0.53 ± 0.20 ^b^	0.01 ± 0.0001 ^b^	***
Zahaf	0.11 ± 0.08 ^ab^	nd	***	0.15 ± 0.01 ^a^	0.006 ± 0.01 ^a^	***
Significance	***	ns		***	*	
	Caffeic Acid					
	UAE	CSE	Significance			
Pizzuta	0.08 ± 0.01ab	nd	***			
Fascionello	nd	nd				
Romana	0.02 ± 0.01 ^a^	nd	***			
Achaak	0.17 ± 0.01 ^b^	nd	***			
Fakhfekh	0.17 ± 0.05 ^b^	nd	***			
Laurane	nd	nd				
Zahaf	nd	nd				
Significance	***					

nd, not detected; mean values followed by different letters within the same column are statistically different at *p* < 0.05 (Duncan’s test); ns, not significant; *** *p* < 0.001; ** *p* < 0.01; * *p* < 0.05.

## Abbreviations

UAE: Ultrasound-assisted Extraction; CSE, Conventional Solvent Extraction; CCD, Central Composite Design; TPC, Total Phenolic Compounds; TCT, Total Condensed Tannins; TFC, Total Flavonoid Content; RSA, Radical Scavenging Activity; GAE, Gallic Acid Equivalent; TE, Trolox Equivalent; CE, Catechin Equivalent.
